# Non-Thrombotic Filling Defects in Cerebral Veins and Sinuses: When Normal Structures Mimic a Disease

**DOI:** 10.3390/neurolint17010009

**Published:** 2025-01-17

**Authors:** Marialuisa Zedde, Rosario Pascarella

**Affiliations:** 1 Neurology Unit, Stroke Unit, Azienda Unità Sanitaria Locale-IRCCS di Reggio Emilia, Viale Risorgimento 80, 42123 Reggio Emilia, Italy; 2 Neuroradiology Unit, Azienda Unità Sanitaria Locale-IRCCS di Reggio Emilia, Viale Risorgimento 80, 42123 Reggio Emilia, Italy; pascarella.rosario@ausl.re.it

**Keywords:** non-thrombotic filling defects, cerebral venous thrombosis, CVT, dural sinus, superior sagittal sinus, transverse sinus, arachnoid granulations, septa, chordae Willisii, MRI, MR venography

## Abstract

Cerebral venous thrombosis (CVT) is a rare and potentially critical cerebrovascular disease involving intracranial dural sinuses and veins. The diagnosis is a stepwise pathway starting from clinical suspicion and employing several neuroradiological techniques, mainly Computed Tomography (CT)-based and Magnetic Resonance Imaging (MRI)-based modalities. The neuroradiological findings, both in the diagnostic phase and in the follow-up phase, may provide some results at risk for misdiagnosis. Non-thrombotic filling defects of intracranial dural sinuses are among them, and the potential sources are artefactual and or anatomical (venous septa and arachnoid granulations). The misdiagnosis of these findings as CVT is potentially linked to dangerous consequences. A potential strategy to avoid this is to increase the knowledge about technical and anatomical reasons for non-thrombotic filling defects of intracranial dural sinuses and their imaging features. The main aim of this review is to address these issues, including the variability of the intracranial venous pathways, providing the solutions for overcoming the above-cited potential misdiagnosis of non-thrombotic filling defects as CVT.

## 1. Introduction

Cerebral venous thrombosis (CVT) is a rare cerebrovascular disease, mainly affecting young women, although it is possible at any age, also by virtue of the associated prothrombotic conditions. In fact, the risk factors are congenital or acquired prothrombotic conditions, sometimes physiological (such as pregnancy and puerperium) and sometimes transitory (e.g., infections), but, especially in the more advanced age groups, neoplasms represent a fairly frequent acquired prothrombotic condition. The clinical manifestations of CVT can be widely variable, from asymptomatic to only isolated, self-limiting headaches up to severe neurological involvement with the development of venous infarction and hemorrhages, as well as intracranial hypertension up to impaired consciousness [1]. In paucisymptomatic cases and in the absence of cerebral parenchymal alterations, the clinical suspicion is sometimes based on weak foundations, cannot count on laboratory support to increase the diagnostic probability, unlike venous thromboembolism in other body sites, and the burden of diagnosis rests almost entirely on the use of neuroradiological techniques, which also have the possibility of misinterpretations and mimicry of CVT. These considerations become fundamental because a misdiagnosis has an impact not only in the case of a false negative result but also in the case of a false positive result, because it conditions the initiation of anticoagulant therapy, with its associated risks (bleeding, thrombocytopenia, etc.). Added to this is the extreme variability of the conformation of the intracranial venous structures, including the main dural venous sinuses, which adds interpretative uncertainty, the possibility of identifying non-thrombotic filling defects in the dural sinuses, and the possibility of artefactual findings in reference to variable velocities and directions of the flow sheets in the intracranial venous district. There are many physiological conditions, sources of artefacts, and conformational variants that can be associated with diagnostic misinterpretation such as CVT, both in the acute phase and in the follow-up phase. In fact, even in sequential controls of the recanalization of previous CVTs, it appears essential to be able to distinguish a thrombotic residue from a non-thrombotic filling defect because it could affect the duration of anticoagulant treatment and the subsequent definition of the risk of CVT recurrence.

Summarizing, CVT is less common than most other types of strokes but can be more challenging to diagnose. CVT refers to dural sinus as well as cerebral vein (cortical and deep vein) thrombosis and is a rare but potentially life-threatening presentation of venous thromboembolism. It is now known to have a more varied clinical spectrum than previously realized. The main pitfalls include clinical suspicion, populations perceived as “at risk” for CVT, neuroimaging techniques and findings (dural sinuses vs. isolated cortical vein thrombosis), and the variability in venous anatomy and hemodynamic issues.

The main aim of this review is to summarize and present the main conditions that can lead to finding of a non-thrombotic filling defect in neuroradiological investigations, primarily Magnetic Resonance Imaging (MRI)-based, with support and differential diagnosis using other techniques, like Computed Tomography (CT)-based and Digital Subtraction Angiography (DSA)-based modalities.

## 2. Cerebral Venous Thrombosis

CVT is a rare but serious condition where a blood clot forms in the cerebral veins, dural sinuses, or both, disrupting the normal drainage of blood from the brain [1] and leading to a variety of symptoms, ranging from headaches to seizures, and, in severe cases, potentially causing brain damage or death [2]. CVT is not a common cause of stroke, but it still accounts for a notable portion of cases: approximately 0.5% to 3% of all strokes. It tends to affect younger individuals, with two-thirds of cases occurring in women, particularly those under the age of 55. The condition can strike at any age but is most frequently diagnosed in young adults and women of childbearing age due to hormonal influences. Pregnancy, oral contraceptive use, and hormone replacement therapies significantly increase the risk of CVT because of their effects on blood coagulation.

Beyond hormonal factors, there are several other risk factors for CVT. Inherited prothrombotic conditions, such as Factor V Leiden or antiphospholipid syndrome, make individuals more prone to forming blood clots. Systemic conditions, like cancer or autoimmune diseases, can also predispose patients to develop CVT. Infections, especially those affecting the head or neck, such as sinusitis, mastoiditis, or even dental infections, are significant contributors to venous thrombosis in the brain. Head trauma or surgery, especially neurosurgical procedures, can also lead to CVT by directly injuring the venous structures of the brain or creating a hypercoagulable state.

The dural venous sinuses are the primary vessels involved in CVT. The SSS is the most commonly affected (25–45%), but other sinuses, such as the transverse sinuses (25–60%), sigmoid sinuses (5–15%), and straight sinus (15–18%), can also be involved. The involvement of more than one sinus is frequent (18–50%) [1]. The thrombosis leads to increased pressure within the venous system and disrupts blood flow. This, in turn, can cause a backflow of blood into the brain, resulting in brain swelling and the formation of edema or hemorrhages.

The symptoms of CVT can be insidious and may evolve over time. The most common presenting symptom is headaches, which occur in up to 85% of patients. The headaches can be severe and are often described as different from typical migraines or tension-type headaches. They may be accompanied by nausea, vomiting, or other signs of increased intracranial pressure. The location of the thrombosis largely determines the nature of other symptoms. Seizures occur in about 40% of patients and can sometimes be the first sign of CVT.

As the clot impedes the flow of blood, neurological deficits may emerge. Patients may develop focal neurological signs such as weakness, numbness, or vision problems depending on the area of the brain affected. An altered mental status, ranging from confusion and disorientation to a coma, may occur in severe cases.

The diagnosis of CVT is often challenging because the symptoms can resemble those of other neurological conditions, such as migraines, brain tumors, or infections. However, imaging plays a critical role in diagnosis, combining CT-based and MRI-based techniques with different sensitivities in different locations (e.g., dural sinuses vs. cortical veins).

## 3. Neuroradiological Techniques: Tips and Tricks

Neuroradiological techniques are the preferred approach for detecting CVT. Non-contrast CT (NCCT) is often the initial imaging modality due to its accessibility and rapid scan times [1,3]. However, its sensitivity is limited, with the classic hyperdense appearance of CVT visible in only about one-third of cases [4]. This limitation makes NCCT suboptimal as a standalone diagnostic tool. To enhance diagnostic accuracy, CT venography is frequently employed alongside NCCT, boasting a sensitivity of approximately 95% [5]. This technique uses iodinated contrast media to identify non-enhancing thrombi within the opacified venous system. However, iodinated contrast agents carry risks of allergic reactions and nephrotoxicity [6]. This limitation has less relevance in an emergency setting, and although theoretically the ionizing radiation involved makes CT less suitable for pregnant patients, the risk of CVT is higher in the third trimester when there is less complaint about the effect of radiation on the fetus.

Magnetic Resonance Imaging (MRI) has emerged as a more robust alternative for CVT diagnosis. MRI offers distinct advantages, including its non-ionizing nature and superior contrast resolution, which allows for the accurate identification and staging of CVT [7,8]. Standard MRI sequences, such as spin and gradient echoes, diffusion-weighted imaging, and inversion recovery, have reported sensitivities and specificities of 79.2% and 89.9%, respectively. Furthermore, magnetic resonance venography (MRV), performed with or without gadolinium, provides detailed evaluations of venous flow. MRV demonstrates a sensitivity of 93.54% and a negative predictive value of 90.9% [9]. However, despite these advantages, MRI is not without limitations. Variations in blood flow, temporal changes in DST, and improper technique selection can lead to misdiagnosis or missed disease.

One of the main explanations for these artifacts is in the features of the most used MRI techniques for visualizing vessels and flow in the vessels. Conventional spin echo imaging (SEI) employs two sequential radiofrequency (RF) pulses to generate a signal. The first, a 90° RF pulse, tips the magnetization vector, followed by a 180° rephasing pulse to correct dephasing effects. Mobile spins, such as those in flowing blood, often evade the rephasing pulse, resulting in the absence of a signal. This phenomenon creates “flow voids” or areas of pronounced hypointensity in patent dural sinuses, depending on the employed sequence [10]. When CVT predominantly consists of red blood cells, the thrombus evolves in a manner akin to a parenchymal hematoma. A hallmark finding on SEI is the loss of these characteristic flow voids, termed “loss of flow void”. This observation should immediately raise suspicion for CVT and prompt a thorough evaluation of the affected dural sinus [11].

Time-of-flight (TOF) imaging utilizes the principle of flow-related enhancement to create contrast between stationary and moving protons. This gradient echo-based sequence suppresses signals from stationary protons through short-interval RF pulses that induce saturation. By aligning the imaging plane perpendicular to the venous flow, TOF imaging captures “bright blood” images. This occurs because unsaturated protons entering the imaging plane generate a strong signal, highlighting flowing blood. To further enhance venous visualization, additional saturation bands are selectively applied to suppress signals from adjacent arterial structures, effectively nullifying their contribution to the image [10].

Phase-contrast (PC) MRV exploits the phase shift that occurs as flowing spins traverse a magnetic gradient. This technique applies bipolar gradients to induce an initial phase shift in mobile protons, followed by subsequent phase restoration. While stationary protons exhibit a net phase difference of zero, moving protons demonstrate non-zero phase shifts due to their spatial displacement [12]. PC imaging uses phase shift data rather than signal amplitude to generate images. Stationary spins, therefore, do not contribute to the image. By employing a flow-encoding gradient or velocity-encoding gradient (VENC), specific flow thresholds can be set to visualize arterial or venous flow. High VENC values highlight arterial flow, while lower VENC values capture venous flow and cerebrospinal fluid (CSF) circulation [10].

Contrast-enhanced MRV leverages gadolinium-based contrast agents, which are paramagnetic and shorten the T1 relaxation times. Once administered, gadolinium increases the intravascular signal intensity, producing a clear “lumenogram.” CE-MRV typically uses a 3D T1-weighted gradient echo sequence with short repetition (TR) and echo times (TE) to enhance the contrast between vessels and surrounding tissues [10]. Techniques such as test bolus and bolus tracking are employed to optimize imaging during the venous phase. CE-MRV provides several advantages, including fast data acquisition, high spatial resolution from thin slices and high matrix settings, and multiplanar reconstruction capabilities. These features are independent of flow dynamics, making CE-MRV particularly useful in diagnosing CVT. CE-MRV has reported sensitivity and specificity rates of 83% and 100%, respectively, for detecting CVT [13].

The main features of MR venography techniques are illustrated in Table 1.

Summarizing the main imaging features in CVT is useful in order to better understand the potential source of a misdiagnosis.

On CT scans, the thrombus can sometimes be directly visualized, especially with the advent of thin-slice CT, which improves the resolution [14]. A dense vessel sign is a characteristic finding in NCCT, where the thrombus appears as hyperattenuating due to the presence of hemoglobin and red blood cells within the clot. This can be seen up to 14 days after the onset of symptoms [15]. The string sign or cord sign is another classic indication of CVT on NCCT. This represents a serpentine or linear hyperdensity within a vein, which suggests the presence of the thrombus. Similarly, a dense triangle sign can also be seen in some cases. The absence of venous filling is another key indicator for CVT in both CT and MRI. In the early stages of CVT, venous obstruction prevents normal blood flow, and this absence can be seen in imaging [15].

Another issue is the imaging of the consequences of venous obstruction on tissue and vascular levels. With both CT and MRI, the effects of venous obstruction can be visualized, such as venous infarction, edema, and hemorrhagic transformation in the affected areas. These may manifest as hypodensities on CT scans or areas of restricted diffusion for MRI. In particular, bilateral hypodensities may be seen when the sagittal sinus or deep cerebral veins are involved. Hemorrhages are observed in about 40% of CVT cases and can include subarachnoid, subdural, or intracerebral hemorrhages, often being associated with the areas of venous infarction or hypodensity. In addition, with CT or MRI, dilated veins can be observed, which indicates increased venous pressure due to the clot blocking normal blood flow.

There are also unique neuroradiological signs. The cashew nut sign (a juxtacortical C-shaped hyperdensity) is a relatively specific indicator for CVT, although it has low sensitivity [16]. Multifocal or bilateral hemorrhages are also commonly seen in CVT cases and can further aid in diagnosis. MRI offers enhanced sensitivity in detecting parenchymal brain lesions due to venous occlusion, such as venous infarcts, which cross arterial vascular territories and may appear bilateral. These infarctions may not conform to the typical wedge-shaped pattern of arterial infarcts.

In MRI, thrombus evolution can be observed over time, as the clot undergoes changes in signal intensity as it ages. Initially, the thrombus may appear as isointense or hypointense on T2 sequences, mimicking the normal flow void seen in venous sinuses [17]. This can make early-stage CVT difficult to diagnose via routine MRI. TOF MRV is another commonly used technique, but absent flow in MRV is not always supported by changes on T1/T2 sequences, leading to potential misdiagnosis. Therefore, combining MRV with other imaging techniques, such as gradient-recalled echo (GRE) or susceptibility-weighted imaging (SWI), can improve the diagnostic accuracy [15,17]. SWI and GRE are particularly useful in detecting thrombosed cortical veins, with near 100% sensitivity and specificity for identifying thrombi in such areas. In more advanced cases, T1-based black-blood imaging is a promising technique that suppresses the signal from flowing blood, making it easier to identify stationary thrombi in veins. This technique has shown promise in enhancing CVT detection, particularly in cases where conventional sequences may miss subtle thrombi.

The signal evolution of a thrombus over time on MRI is summarized in Table 2.

A recent meta-analysis of studies assessing the diagnostic performance of CT and MRI in CVT [14] showed the following:

- CT scans had a sensitivity of 0.79 and specificity of 0.90 in detecting CVT.
- MRI sequences, including conventional T1/T2 imaging and MRV, had a sensitivity of 0.82 and a specificity of 0.92.

MRI is particularly superior in detecting parenchymal brain lesions due to venous occlusion and venous infarctions, making it a crucial tool in diagnosing CVT when CT findings are inconclusive [19].

Despite the advancements in imaging techniques, there are some challenges. Imaging findings, particularly on CT scans, can sometimes be subtle or difficult to interpret, especially in the early stages or if there are atypical clinical presentations. Flow gaps on MRV or subtle signal changes on T2-weighted sequences may not always correlate with actual thrombus formation, necessitating complementary imaging techniques like SWI or gradient-recalled echo.

For diagnosing CVT, CT venography (CTV) and MRV are the optimal imaging tests. These methods provide detailed visualization of the cerebral venous system and help confirm the presence of thrombi. DSA is typically reserved for cases where invasive treatments, such as thrombectomy or thrombolysis, are being considered.

CTV is a valuable diagnostic tool for depicting both the superficial and deep cerebral venous systems. It provides a clear image of thrombi, which typically appear as filling defects, such as the characteristic “empty delta sign” in the superior sagittal sinus. This sign is particularly helpful in distinguishing a thrombus from other anatomical features, like arachnoid granulations, which might otherwise be confused with thrombotic material. CTV offers high sensitivity and specificity for detecting CVT, often outperforming other imaging modalities, including DSA, in terms of diagnostic accuracy. However, CTV tends to have lower sensitivity for detecting thrombosis in cortical veins compared to MRI, particularly in subtle cases of cortical vein thrombosis [17].

MRV can be performed using various techniques, as previously outlined:

- TOF MRV works by generating images based on the natural flow of blood and is particularly useful in cases where contrast agents cannot be used (e.g., in pregnant or breastfeeding patients or those with severe renal failure).
- PC MRV measures blood flow velocity and is more accurate than TOF in certain cases but is less commonly used due to its complexity, longer acquisition times, and operator dependency.
- CE MRV is more sensitive, allowing for more precise detection of thrombi, especially within smaller veins. This technique is particularly helpful in distinguishing between low-flow states and hypoplastic sinuses (flow gaps), which can be challenging to differentiate without contrast.

When DSA served as the reference standard, contrast-enhanced MRI outperformed non-contrast-enhanced MR sequences, achieving a sensitivity and specificity of 83% and 100%, compared to 8–51% and 80–93%, respectively [17]. A recent meta-analysis summarizing the diagnostic accuracy of MRV techniques (both contrast- and non-contrast-enhanced) for CVT confirmed the superior performance of CE MRV over non-contrast-enhanced TOF and PC MRV [14]. CE MRV offers comparable sensitivity and specificity to CTV but with the added benefit of better characterization of complex venous structures. It is particularly useful in identifying cortical vein thrombosis, and when combined with gradient-recalled echo or susceptibility-weighted imaging (SWI), it provides an even more accurate diagnosis of CVT, particularly in cases involving small veins or cortical venous thrombosis.

CTV is highly effective in evaluating the superficial and deep venous systems, providing clear images of the major sinuses. Relatively old studies highlight CTV as a highly reliable alternative to DSA in diagnosing CVT with a sensitivity and specificity near to 100% [17]. However, the strength of this evidence remains limited due to the small sample sizes (<100 patients), the observational nature of the studies, and significant risk of bias [17]. When compared to MRI, CTV demonstrated a sensitivity and specificity of 100% for diagnosing CVT [17]. As a limitation, CTV has limited sensitivity for cortical vein thrombosis and may not always detect subtle cases. MRV, especially with contrast, offers excellent sensitivity and specificity, including better characterization of small venous structures. However, it may be subject to flow artifacts and requires more advanced techniques, such as GRE or SWI, for complete assessment. Non-contrast TOF MRV is a preferred option when gadolinium use is contraindicated but may be less sensitive for detecting a slower venous flow compared to contrast-enhanced techniques.

For accurate detection and confirmation of CVT, the following hold true:

- CTV is optimal for a first-line imaging modality, particularly for evaluating the major venous sinuses.
- CE MRV is highly recommended for a better assessment of smaller veins and cortical vein thrombosis, with TOF MRV being a good alternative in situations where contrast cannot be used.
- For diagnosing cortical vein thrombosis, newer imaging methods like SWI and GRE sequences should be employed alongside MRV for a comprehensive diagnosis. These techniques have shown higher diagnostic accuracy in identifying thrombosed cortical veins.

## 4. Non-Thrombotic Focal Filling Defects in the Intracranial Veins

### 4.1. Normal Structures in the Intracranial Dural Sinuses

The anatomical structure of the dural sinuses is complex, and in most cases, they are not simple channels but were instead formed via the fusion and regression of paired structures in to the dural folding, with a final pattern of internal irregularities, septations, duplications, and prominent arachnoid granulations. The superior sagittal sinus (SSS) and transverse sinus (TS) are the most easily recognizable in these features.

The SSS is a structure of remarkable complexity, being shaped like a triangular cross-section. Within its interior lie the openings of the superior cerebral veins and the distinctive projections of arachnoid granulations. Its architecture is traversed by numerous fibrous bands, known as the chordae Willisii, which add to its intricate design. Small orifices within the sinus allow for communication with irregular venous lacunae embedded in the dura mater nearby. These lacunae are fascinating in their variety and arrangement. Typically, there are two or three small frontal lacunae, larger parietal lacunae, or intermediate-sized occipital lacunae positioned along each side of the sinus. In older individuals, these lacunae often merge to form a single, elongated structure on each side, intricately crossed by fine fibrous bands and hosting numerous arachnoid granulations. The lacunae serve a vital function, draining the diploic veins within the spongy bone of the skull and the meningeal veins of the dura mater. Their complexity is further highlighted by their often-plexiform nature, resembling a network of interconnected venous spaces rather than simple cavities. Around the SSS, plexiform arrays of small veins extend toward adjacent sinuses, including the transverse and straight sinuses. From these areas, ridges of “spongy” venous tissue frequently project into the lumina of the superior sagittal and transverse sinuses, contributing to the rich and dynamic architecture of this venous system. This intricate design underscores the SSS’s vital role in the venous drainage of the brain and the fascinating complexity of its connections and supporting structures.

When evaluating the dural sinuses for thrombosis, several anatomical and pathological entities can be observed on imaging, including arachnoid granulations, intrasinus septa (fibrous bands), and developmental variations such as hypoplasia or aplasia of the dural sinuses. Among these, arachnoid granulations are commonly encountered in the SSS, with imaging studies reporting their presence in 0.3% to 55% of cases [20]. These structures tend to become more prominent with advancing age and may indent the overlying calvarial bone, which can be visualized through imaging modalities. High-resolution, 3D contrast-enhanced, magnetization-prepared rapid acquisition gradient echo (MPRAGE) imaging is a valuable tool for identifying intracranial dural sinus thrombosis. This technique utilizes a pulse sequence with a section thickness of 1.25 mm to produce detailed 3D datasets. In cases of intradural thrombosis, the affected areas appear hypo- or isointense relative to the brain parenchyma and exhibit a high contrast against the bright signal of flowing blood in MPRAGE images. Thromboses are characterized by irregular margins, an expansion of the sagittal sinus, and, often, extension into adjacent sinuses. Arachnoid granulations (AG), in contrast, exhibit distinct imaging characteristics. These granulations are isointense to cerebrospinal fluid (CSF), maintain sharp margins, and do not cause widening of the dural sinus or extend into neighboring sinuses. This clear differentiation is crucial for accurate diagnosis and management.

In terms of diagnostic performance, 3D contrast-enhanced MPRAGE imaging demonstrates the highest sensitivity (83%) and specificity (99%) for detecting dural sinus thrombosis. This significantly outperforms other imaging modalities, such as 2D TOF MR venography (sensitivity 51%; specificity 92%) and T1-weighted MRI (sensitivity 33%; specificity 84%). These findings highlight the critical role of advanced imaging techniques in accurately diagnosing dural sinus thrombosis and differentiating it from normal anatomical structures or variations [20].

#### 4.1.1. Septations

The chordae Willisii, first described by Thomas Willis in 1664, are fibrous bands located within the SSS and the TS of the dura mater. These structures play a critical physiological role and exhibit significant variation in morphology and distribution.

The chordae Willisii can be classified into three main types (Table 3):

On average, there are 17 chordae Willisii per sinus, although this number can range from 11 to 25. Each sinus has unique configurations, as no two specimens have identical arrangements of these structures [21,22]. The highest density of chordae is located in the parietal region, where the middle segment of the SSS is rich in bands, bridges, and cusps. Fewer chordae are observed in the anterior portion.

The chordae Willisii serve several physiological purposes:

- Venous flow regulation: by covering venous orifices partially, valvelike chordae prevent retrograde blood flow;
- Structural support: trabecular and longitudinal chordae maintain the integrity of the sinus lumen and prevent collapse under pressure fluctuations;
- Flow optimization: the arrangement of trabecular projections and asymmetry ensures optimal blood flow dynamics, particularly in the right-sided dominance of the SSS.

**Figure 1:**
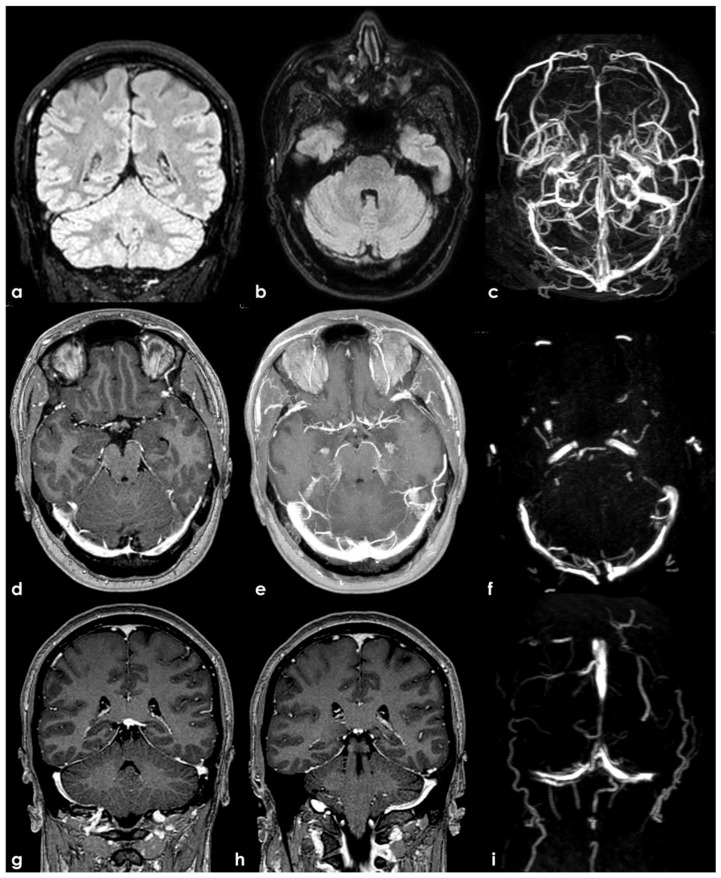
An example of TS septations. The panels (**a**,**b**) show, in coronal and axial planes, respectively, a hyperintense crescent on the right transverse sinus at the transverse–sigmoid sinus transition. In panel (**c**), reconstructed PC MRV shows a linearly interrupted flow signal in the right TS and a focal interruption in the distal third of the left TS. In panel (**d**,**e**), a 3D CE-T1W sequence reconstructed in the axial plane with the MRP protocol (source image in panel (**d**) and MIP formatted image in panel (**e**) show a linear hypointense structure within both TSs (better imaged in source images), as a flow separator (panel (**f**,**i**), source image of PC MRV in axial and coronal planes, respectively). In the coronal plane (panel (**g**,**h**)), these findings are followed along the transverse–sigmoid sinus transition.

**Figure 2:**
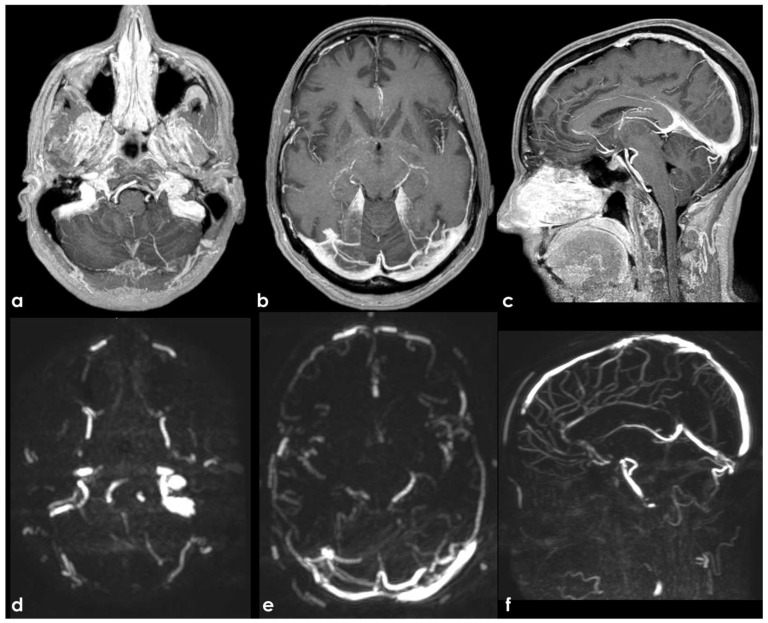
An example of TS septations. Panels (**a**–**c**) show the 3D CE-T1W sequence reconstructed in the axial plane (**a**,**b**) and sagittal plane (**c**) with the MIP/MRP protocol. A linear hypointense structure within both TSs as a flow separator is visible, and it can be followed along the transverse–sigmoid sinus transition, also imaged in PC MRV (panel (**d**–**f**), source images of PC MRV in axial and sagittal planes). In addition, PC-MRV reconstructed in the sagittal plane (panel (**f**)) confirmed the change in size along the course of the SSS, as imaged in panel (**c**).

The chordae Willisii develop during the fetal period and increase in number and complexity with age. Animal models, such as those conducted on bovine sinuses, reveal consistent chordae formations, offering insights into their growth patterns and functions [24,25]. Cadaveric studies have significantly contributed to understanding the diversity and role of the chordae Willisii. Their unique structural and functional attributes underscore their importance in venous drainage and intracranial pressure regulation. Insights into these structures provide a foundation for diagnosing and managing conditions affecting the dural venous sinuses, such as thrombosis or structural anomalies, as illustrated in Figure 1 and Figure 2.

#### 4.1.2. Arachnoid Granulations

Among the several anatomical structures and variations that may be encountered in the imaging evaluation of the dural sinuses, there are arachnoid (Pacchionian) granulations. AGs are protrusions of the arachnoid mater into the dural sinuses. These are normal structures that can be observed in two-thirds of cadaveric specimens, primarily in the superior sagittal sinus and transverse sinus [26,27,28]. The reported frequency of AGs on imaging varies widely, ranging from 0.3% to 55%, depending on the population and imaging technique used. This wide range may be influenced by the resolution of imaging modalities and the population studied [26,29,30].

On imaging, these granulations appear as small, nodular protrusions into the sinus lumen. While they are often benign and asymptomatic, their appearance can sometimes be mistaken for pathological processes such as thrombi.

Interestingly, SGs are rarely reported in the superior sagittal sinus or straight sinus in imaging studies, likely due to limitations in visibility on conventional axial MR and CT scans. The SSS is difficult to visualize in these imaging planes, particularly due to volume averaging, where the sinus may blend with surrounding structures, such as the cranial vertex, which reduces the resolution.

A relatively early study investigated the MR imaging characteristics of AGs using a 3D contrast-enhanced MPRAGE sequence on a 1.5 T system in 100 subjects with patent dural sinuses [20]. This study identified 433 round, oval, or lobulated focal filling defects in 90 patients. Additionally, curvilinear septa were observed in 92 patients. Among these cases, 69 patients had round, oval, or lobulated defects in the transverse sinus (TS), 59 in the SSS, and 47 in the straight sinus. Nearly all defects, except for two, were isointense to CSF across all imaging sequences, leading to their classification as AGs. Of the 431 identified AGs, 233 (53.8%) were located in the SSS, 122 (28.1%) in the TS, and 76 (17.6%) in the straight sinus. In 96% of cases (414 instances), one or more veins were observed entering the AGs. Notably, the focal filling defects caused by AGs are often misinterpreted as acute CVT. Conversely, defects caused by curvilinear septa are more frequently misdiagnosed as subacute or partially recanalized thrombosis. An intriguing finding was the identification of two filling defects that were isointense to brain parenchyma across all imaging sequences, presumed to represent brain tissue invaginations into the dural sinus. While rare, this phenomenon of heterotopic brain tissue invaginating into a sinus must be considered in the differential diagnosis of focal filling defects within dural sinuses. Such cases can mimic thrombi on imaging modalities like CT or MRI due to their similar appearance. A notable report described a case where brain tissue invagination into a dural sinus was initially misdiagnosed as a thrombus [31]. This condition occurs when heterotopic brain tissue, which is normally absent in the venous system, protrudes into the sinus, causing irregularities or anomalies that may lead to diagnostic errors. Careful differentiation of these anomalies is crucial to avoid misdiagnosis of CVT. The authors attributed the higher detection rate of AGs in their study compared to earlier research to the use of an advanced MRI technique: a heavily T1-weighted imaging method with a high signal-to-noise ratio. This technique eliminates interference from slow or in-plane flow, enabling the identification of AGs as small as 1–2 mm [20]. AGs in the transverse sinus (TS) were particularly evident on both conventional MR images and 3D contrast-enhanced MPRAGE sequences due to their relatively large size (mean diameter of 4 mm) and the distinct signal intensity contrast with surrounding structures [26]. Most AGs in the TS were located in its lateral third and at the TS–sigmoid sinus junction, where the vein of Labbé and cerebellar veins enter, consistent with previous findings [26,29,32].

On 3D contrast-enhanced MPRAGE images, numerous smaller granulations (<2 mm) were commonly detected at the anterior and superior portions of the superior sagittal sinus (SSS), particularly near sites where veins join the dural sinus. These granulations typically invaginated into the lateral and inferior surfaces of the SSS. Larger, solitary granulations were occasionally found in the posterior or caudal SSS, approximately 2.5 cm rostral to the torcular Herophili.

**Figure 3:**
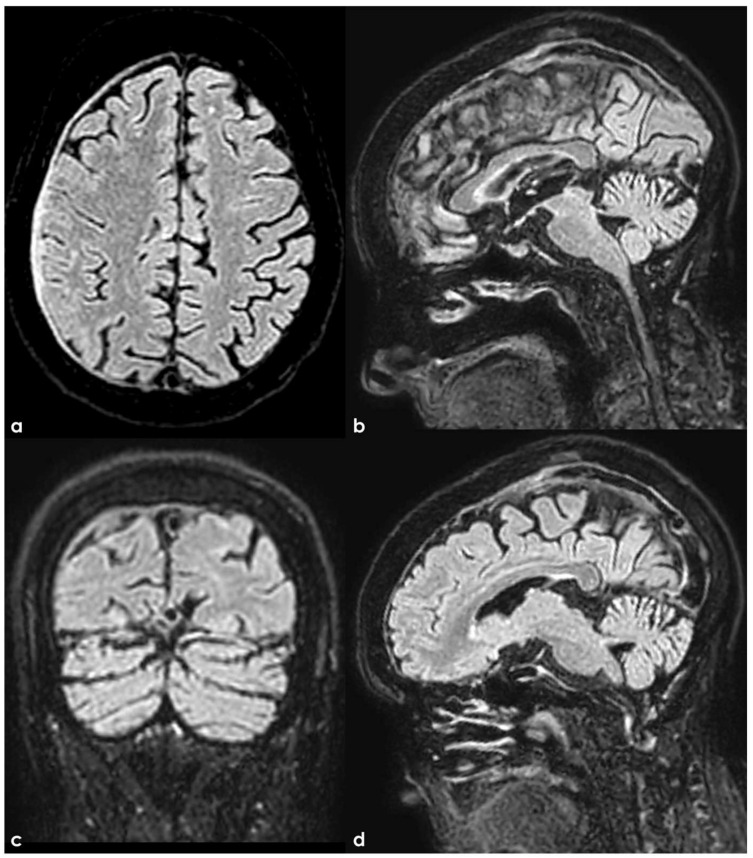
An example of SSS septations and associated AG. Here, 3D-FLAIR images are reconstructed in MPR in the axial (panel (**a**)), sagittal (panel (**b**,**d**)), and coronal (panel (**c**)) planes. The section of SSS in panels (**a**,**c**) seems to have a double lumen, and in panels (**b**,**d**), a long tortuous hyperintense septation within the SSS is recognizable starting near to a discrete rounded AG, with hyperintense boundaries and filling content with the same signal as CSF.

**Figure 4:**
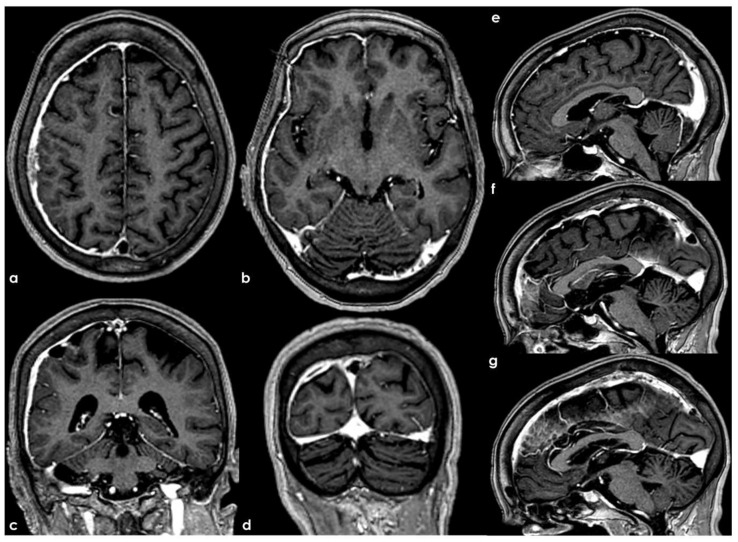
Same subject as in Figure 3 imaged using 3D CE-T1WI. The source images were reconstructed in the axial (panel (**a**,**b**)), coronal (panel (**c**,**d**)), and sagittal (panel (**e**–**g**)) planes. The post-contrast images better depicted the double-channel appearance of the SSS and the large AG already proposed in the previous figure. In the left transverse sinus, few small focal filling defects are shown, suggestive for AGs.

**Figure 5:**
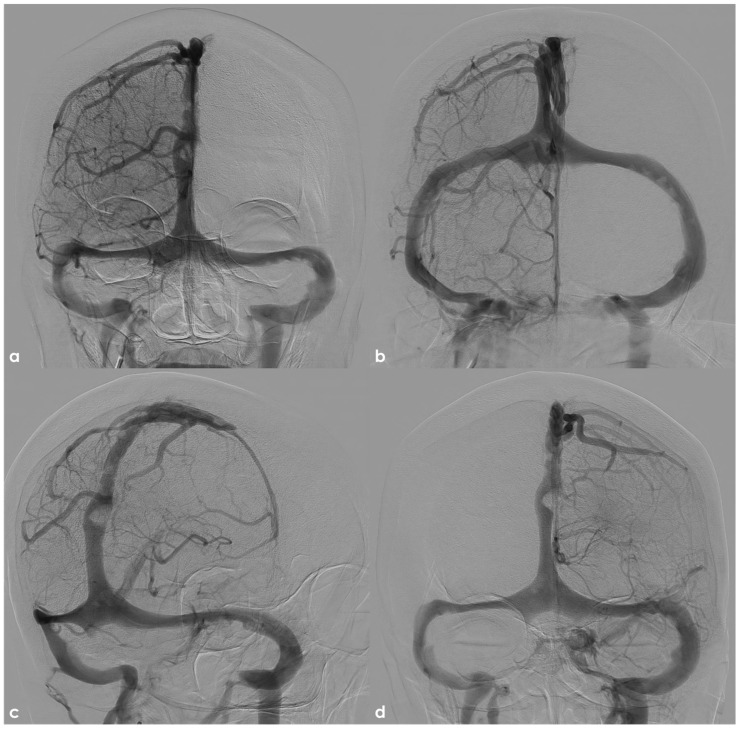
DSA in the venous phase (same subject as in Figure 3 and Figure 4) in posterior–anterior (panel (**a**)), semiaxial (panel (**b**)), oblique (panel (**c**)) from right ICA injection, and posterior–anterior with left ICA injection (panel (**d**)). Some irregular filling defects are evident in the SSS and in the left TS, with a rounded appearance when AGs and a tiny serpentine pattern when septation in the SSS.

One notable structural characteristic observed was that approximately 96% of granulations (414 out of 431) were associated with veins entering them along the internal sinus wall, specifically at the orifices leading into the subarachnoid space. This finding aligns with observations from cadaveric studies [27]. An example is illustrated in Figure 3, Figure 4 and Figure 5.

A distinct subtype of AGs, referred to as “giant” AGs, has been observed, particularly favoring the posterior third of the SSS [33]. Their imaging features include a signal intensity resembling CSF, an extension of the subarachnoid space into the granulation due to a focal defect in the sinus dural margin, and the presence of intrinsic vessels, likely displaced cortical veins or sinus septations. These AGs are often associated with nearby cortical veins and are typically located near the lambda or at the posterior edge of the parieto-occipital sulcus. They consistently lack brightness on FLAIR imaging, show no diffusion restriction, and do not exhibit intrinsic mass-like enhancement. In some instances, these features have led to their misdiagnosis as CVT.

**Figure 6:**
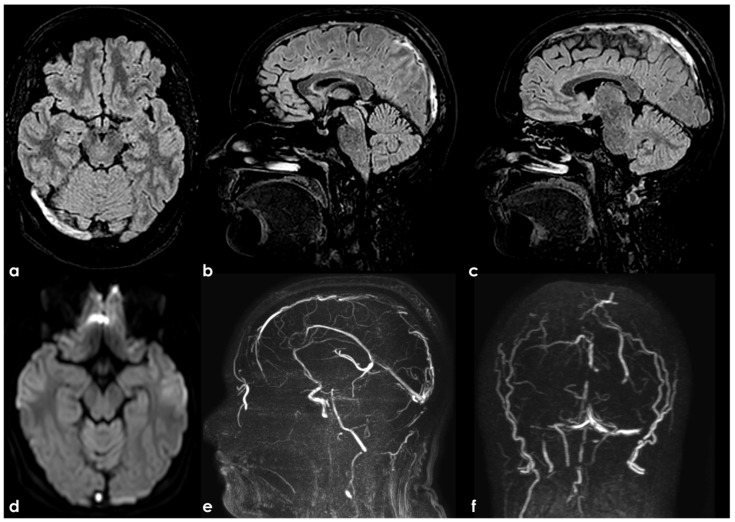
Brain MRI and PC-MRV in a woman with subacute CVT involving the SSS and TS on both sides, sparing the torcular. Axial and sagittal FLAIR (panels (**a**) and (**b**,**c**), respectively) shows the spontaneous hyperintensity of the right TS and SSS. The clot in the right TS shows a focal hyperintensity in diffusion-weighted imaging (DWI) (panel (**d**)). PC-MRV shows multiple irregular flow defects in the SSS and TS on both sides (panels **e**,**f**).

Additionally, calvarial remodeling is a helpful diagnostic clue for AGs. This remodeling is frequently seen near the SSS or lateral lacunae [27] but has also been reported in cases involving AGs adjacent to the TS and temporal bone [34,35]. In Figure 6, an example of a thrombotic filling defect is illustrated, as a comparison.

### 4.2. Artifacts

False positive and false negative errors are common during MRI for diagnosing CVT. In this review, the false positive findings are mainly considered.

SEI may be affected by the following pitfalls:

- Apparent “flow void”: In acute thrombus formation (0–5 days), the presence of paramagnetic deoxyhemoglobin renders the thrombus isointense on T1-weighted (T1W) images and hypointense on T2-weighted (T2W) images. This can mimic the flow void typically seen in patent sinuses on SEI [36]. To avoid misinterpretation, luminal signals should also be evaluated using GRE or SWI. These sequences are highly sensitive to magnetic field inhomogeneities caused by the paramagnetic thrombus, appearing as hypointense “blooming” artifacts. Any such susceptibility signals within dural sinuses should raise suspicion for CVT and prompt confirmatory venography [3].
- Loss of flow void: As a thrombus transitions into the subacute phase (6–15 days), deoxyhemoglobin is replaced by methemoglobin, leading to hyperintensity on both T1W and T2W images. This appearance, termed “loss of flow void,” can resemble slow venous flow [37]. Slow flow may mimic a CVT signal. It usually affects isolated venous segments and appears isointense on T1W images. Protons in slow-moving blood are rephased by the 180° RF pulse, creating a bright intraluminal signal. PC-MRV, due to its insensitivity to T1 signal characteristics and ability to detect all flowing spins within a given VENC, allows for reliable differentiation between slow flow and subacute CVT, in particular using lower VENC settings to improve the accuracy [38,39]. In addition, in slow flow states, blooming artifacts, due to high paramagnetic properties of methemoglobin in GRE sequences, are absent [10,33]. Subacute thrombi often demonstrate restricted diffusion on diffusion-weighted imaging (DWI), reflecting their dense microstructure [40].
- Entry Slice Phenomenon (ESP) occurs when a paradoxically bright signal is observed in the initial slices of T1W sequences. It results from unsaturated or “relaxed” spins entering the imaging slice, contrasting with stationary spins saturated by the short TR. This effect diminishes across subsequent slices as the blood becomes progressively saturated by rapid RF pulses [10]. ESP is confined to the first few imaging slices and fades in subsequent slices. PC-MRV can confirm flow independently of T1 signal characteristics. Reacquiring images in a different plane can help differentiate true thrombi from artifacts: true thrombi retain their bright signal irrespective of orientation, whereas ESP disappears [41].

MRV, too, may be subject to artifacts and physical phenomena being known in order to avoid misdiagnosis. Among these, the “shine-through” phenomenon in TOF-MRV imaging is related to the brief TR interval functioning as a surrogate for the T1W sequence. Tissues with inherent intravoxel T1 shortening tend to exhibit a “shine-through” effect, where they appear abnormally bright on TOF imaging. This includes methemoglobin present in subacute CVT, which, due to its T1 hyperintensity, causes an artifact that results in an exaggerated signal on TOF-MRV. Although the signal is typically less intense than that of flowing blood, it may still be mistaken for patency. To accurately assess for thrombosis, it is crucial to examine GRE/SWI images for any blooming or susceptibility artifacts and to employ CE-MRV with subtraction techniques. Additionally, PCMRV is invaluable in these cases because it is not affected by T1-shortening effects, providing a more reliable assessment of venous patency [10,36]. Another artifact is the in-plane saturation (IPS) one. Flow signals on TOF images are brightest when the blood flow is perpendicular to the imaging plane. However, if the dural sinuses are aligned parallel (“in-plane”) to the imaging sections, the spins become saturated and fail to generate signals, leading to signal loss. This phenomenon, which occurs in otherwise patent dural sinuses, is referred to as “in-plane saturation.” It is important to distinguish this from actual CVT to avoid diagnostic errors [42,43]. Recognizing the acquisition orientation is critical for detecting IPS. For instance, coronal TOF venograms are useful for evaluating the midline sinuses, but IPS may still be problematic, particularly at the torcular and transverse sinuses. To confirm this, switching the acquisition to an orthogonal plane can eliminate IPS-induced signal loss. No single rectilinear plane can be perfectly perpendicular to all dural sinuses in one acquisition [44].

While TOF-MRV can detect slow flow, it has a minimum flow threshold, below which spins are saturated by the short RF pulse interval, leading to signal loss, or “flow gaps”. This effect is particularly pronounced with hypoplastic dural sinuses and can be exacerbated by coplanar acquisition. A study by Ayanzen et al. [44] found that 31% of non-dominant transverse sinuses exhibited flow gaps, but none were seen in dominant transverse sinuses or the rest of the dural venous system. Thus, confirming the absence of thrombosis is essential before diagnosing a flow gap [44]. The absence of thrombus-related signals, along with the use of CE-MRV (which is independent of flow dynamics), is crucial for confirming sinus patency.

PC-MRV detects flow information through phase shifts in protons within a selected VENC. However, flow velocities outside of this range are not captured, causing signal loss that can mimic an occlusion. This issue often occurs in the lateral third of the TSs, especially when local flow turbulence is caused by AGs or stenosis in idiopathic intracranial hypertension (IIH). Fera et al. studied this signal loss in IIH and noted that lower VENC values (15 cm/s) were insufficient to detect high-velocity turbulent flow. Increasing the VENC to 40 cm/s resolved the signal gap. Therefore, selecting an appropriate VENC is crucial for distinguishing stenosis from thrombosis [39]. Given that CE-MRV is independent of flow and sinus geometry and considering its high spatial resolution, it serves as a valuable complement to PC-MRV when the cause of signal loss remains unclear [39].

CE-MRV presents several challenges due to potential misinterpretations. During the chronic phase of CVT, the thrombus typically exhibits T1 isointense and T2 hyperintense signals. Histologically, this stage involves fibroblast proliferation and the development of capillary networks within the thrombus, gradually transforming it into a fibrotic mass with multiple endothelial-lined channels. These channels can exhibit intense enhancement on contrast imaging, which may misleadingly indicate normal sinus patency. Furthermore, chronic dural sinus thrombosis may lack susceptibility or blooming artifacts, as macrophages clear denatured hemoglobin from the thrombus during its organization, reducing the reliability of certain imaging sequences.

**Figure 7:**
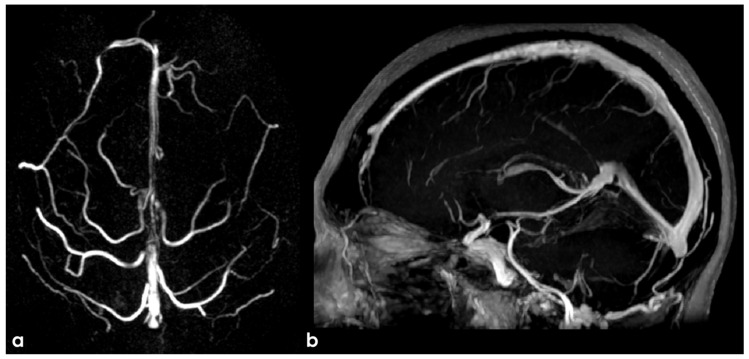
PC MRV reconstructed on a superior–inferior view, showing the SSS with progressive decrease in the flow signal in the middle segment (panel (**a**)). Also, 3D CE-MPRAGE reconstructed in a sagittal view did not confirm the change in flow signal in the SSS, helping to exclude CVT. In panel (**b**), at the junction between the Galen vein and the straight sinus, a focal filling defect is imaged, suggesting an arachnoid granulation.

**Figure 8:**
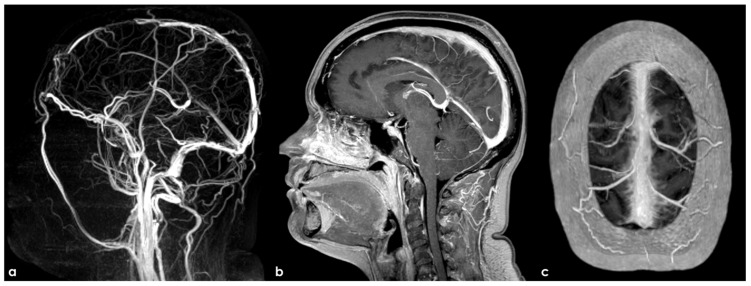
Similar findings as in Figure 1. In panel (**a**), PC MRV reconstructed in a lateral view shows the decrease in the flow signal in the anterior segment of the SSS, not confirmed in CE-MRV reconstructed in sagittal plane (panel (**b**)). The same sequence in an axial plane along the SSS course confirmed the normal contrast filling of the venous structures (panel (**c**)).

**Figure 9:**
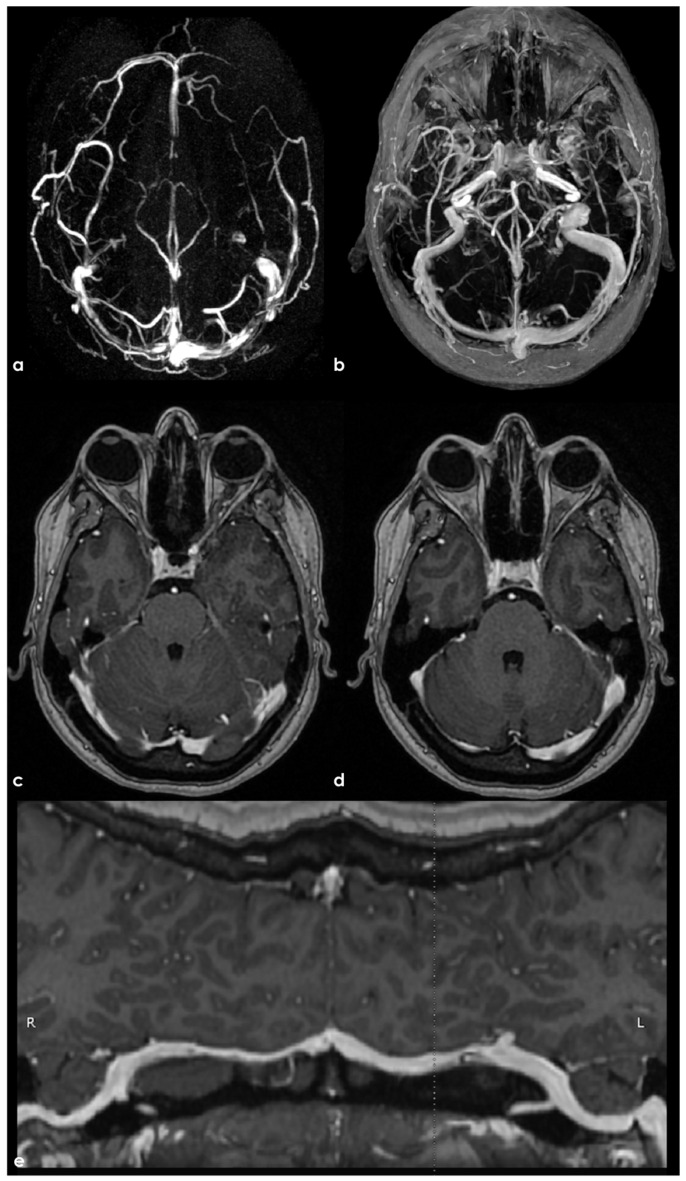
Another comparison between 3D TOF-MRV and CE-MRV (3D MPRAGE) in depicting the patency of the transverse sinus on both sides. Panel (**a**) shows TOF-MRV reconstructed in an axial plane with a significant decrease in the flow signal in the proximal and middle portions of the right TS and on the proximal and distal segments of the left TS. The same view using CE-MRV did not confirm these findings (panel (**b**)). MPRAGE (panels (**c**–**e**)) allowed us to identify a tiny longitudinal septum in the right and left TSs and a focal filling defect in the distal third of the left TS, suggesting the presence of a small AG.

A study by Leach et al. reported susceptibility artifacts in 90.6% of CVT cases within the first 7 days but in only 23.3% of cases older than 8 days [45]. In such scenarios, unenhanced MR venography is often adequate for evaluating the sinus lumen [26,46]. Another cause of non-thrombotic filling defects are AGs. They are invaginations of arachnoid matter into venous sinuses through dural apertures, serving as sites for CSF resorption. They most commonly occur along the lateral portion of the TSs (with left-side predominance) and the SSS [46]. On unenhanced T1W images, AGs appear isointense to hypointense, and they are consistently hyperintense on T2W images. FLAIR imaging may not fully suppress the CSF signal, resulting in partial suppression due to altered CSF flow. AGs vary in size, and large ones may resemble a thrombus. To differentiate these filling defects from thrombosis, key imaging features include their focal, well-defined, round–oval, or lobulated shape and the absence of susceptibility artifacts on GRE/SWI sequences. AGs are never hyperintense on T1W images. Another common, although not always present, finding is the presence of cortical veins entering the granulations. Liang et al. reported that 96% of AGs (414 out of 433) contained one or more veins [20]. Fibrotic septations, often found in the straight and TSs, are thin linear filling defects aligned along the sinuses’ long axis. Unlike defects in partially recanalized CVT, septations have sharp, well-defined margins. Occasionally, AGs may be found at the ends of septations [20,47]. Figure 7, Figure 8 and Figure 9 show different MRV findings, comparing TOF and CE-MRV.

### 4.3. Main Anomalies/Variations

The intracranial venous circulation, with particular reference to the main dural sinuses, is characterized by a wide variability of conformation. Hypoplasia or aplasia of entire segments is quite common and often asymmetric. Sometimes, in the clinical context with moderate or high probability, the absence of flow that can be sampled on a segment of a dural venous sinus can enter into the differential diagnosis between thrombotic occlusion and aplasia. In fact, the dural venous sinuses evolve from a complex venous network, leading to various anatomical variations [46]. A lack of familiarity with these variations can result in misdiagnosis and complicate interventional neurosurgical procedures. Therefore, it is crucial to understand not only the typical anatomy of the dural sinuses but also the possible variants. Listing all the possible conformational variants of the intracranial venous sinuses is beyond the scope of this review. The selected information, therefore, refers to those of greatest relevance in the differential diagnosis between an anatomical anomaly and a pathological condition (CVT in this case) and focuses on the main dural sinuses most involved in CVT.

The SSS has a diameter ranging from 3.0 to 4.5 mm [48], with its length varying from 24 to 27 cm in a single report [49]. In one study of 100 patients, 7% had hypoplasia of the rostral superior sagittal sinus [50]. Browder and Kaplan observed that the length of rostral sinus atresia ranged from 1 to 9 cm [51], and the complete absence of the SSS has also been reported [52,53]. Bisaria documented splitting of the SSS into two or three channels, either with or without partition [48,54]. The SSS typically drains into the torcular Herophili, superior jugular bulb, or the left or right TS.

The inferior sagittal sinus can also be absent, with Saxena et al. reporting an absence in 1 out of 76 cases [51,55].

The torcular Herophili is a region where several dural sinuses converge, and variations in its development have been extensively documented. Labbé [56] quoted Hallet’s first description [57] of an absent torcular Herophili. Several classifications of the variant patterns of the SSS, straight sinus, and TS confluence were proposed. They are summarized in Table 4.

Woodhall further suggested various TS draining patterns, resulting in distinct forms of the torcular [57]. In the plexiform type, both the SSS and straight sinuses may drain equally into both TSs, or one sinus may drain exclusively into one TS while the other drains into the opposite sinus. Additionally, Bisaria [48,53] observed rare variations, including double straight sinuses merging to form one sinus, which drains into the left TS; a TS originating from a tentorial vein; a circular defect in the roof of a TS; and drainage of the tentorial vein into the torcular Herophili. Surendrababu et al. [63] found that 39% of the SSS drained into the right TS, 15% into the left TS, and the remaining 46% into the torcular Herophili. Curé et al. [64] also observed a variation where the basal vein of Rosenthal drained near the torcular Herophili. Browder and Kaplan [51] noted triangular, figure-eight, and circular configurations of the torcular Herophili.

The TS, too, shows several variations. Hollinshead reported hypoplasia or absence of the TS [52]. Knott noted that the right TS is generally larger than the left, with the right TS being four times more commonly larger [52]. However, the left TS may occasionally be longer than the right. Browder and Kaplan observed that 33% of specimens exhibited right-sided dominance of the TS, while 16% showed equal dominance, and 8% demonstrated left-sided dominance [51]. In addition, Browder and Kaplan observed cases where the distal 1–3 cm of the SSS was doubled, with the smaller channel giving rise to a small TS [51]. They also reported narrowing of the TS lumen, caused by the presence of a vascular mesh. Another variation noted was the presence of numerous connections joining the torcular Herophili to the TS. Aplasia and hypoplasia of one TS are relatively common, with hypoplasia being more prevalent. These variations are typically seen on the left side, although they can occur on the right as well. In cases of complete aplasia, both the SSS and straight sinuses drain into the contralateral TS [65]. Rare instances of the absence of one or both sinuses have also been reported [66]. Browder and Kaplan [51] studied 422 specimens and found developmental abnormalities of the proximal TS in 13 cases. Narrowing was observed on the right side in five cases, the left side in seven cases, and both sides in one specimen. In the latter case, the SSS continued as a large occipital sinus, joining the marginal sinus. They also reported atresia of the TS in 11 specimens and the doubling of parts of the sinus in 14 specimens. Hahn and Streit [67] observed TSs separated by a fibrous septum or bony ridge.

Williams and others [65] reported unilateral absence of the sigmoid sinus. Rutting described a diverticulum in the sigmoid sinus, while Furstenberg reported a sigmoid sinus ending in a blind pouch, draining through a large mastoid foramen [68,69]. Hypoplasia or total aplasia of the sigmoid sinus is often observed on the left side. In cases of both TS and sigmoid sinus absence or hypoplasia, the superior petrosal sinus may pass directly through the mastoid foramen, with a prominent inferior petrosal sinus [65]. Surendrababu et al. [63] found that 6 patients had a hypoplastic right sigmoid sinus, while 19 had a hypoplastic left sigmoid sinus. Duplication of the sigmoid sinus is rarer than that of the transverse sinus, with the two channels often separated by a fibrous or bony septum [65]. In very rare cases, the sigmoid sinus may take an unusual anterior course, reaching the posterior wall of the external auditory canal [70].

Saxena et al. [71] found that 13.85% of straight sinuses were doubled in 43 cadavers, with 9.3% in the median position and 4.65% in the paramedian position. Magden [72] found a 26.7% incidence of straight sinus duplication in cadavers, and in 3.5% of cases, the sinus was triple (either median or paramedian). The length of the straight sinus ranges between 4 and 7 cm [55]. In the absence of the inferior sagittal sinus, the straight sinus is formed by the continuation of the great cerebral vein, and it may also communicate with the occipital sinus, cavernous sinus, or right TS [55,73]. Hasegawa et al. [74] examined 108 control cases and found that the straight sinus had an average length of 51 ± 5.2 mm. The straight sinus can be completely or partially absent. Van den Bergh et al. [47] reported a congenital case of an absent straight sinus in a patient with Aarskog syndrome. Knott [52] described a similar instance. Hsu and Chalopka [75] reported a case where the persistent falcine sinus connected the vein of Galen to the posterior third of the SSS, with only the proximal end of the straight sinus being absent. The distal end of the straight sinus drained the occipital lobe and superior cerebellum. Kim and Lee [76] reported two cases of persistent falcine sinus with complete absence of the straight sinus. Ryu et al. [77] examined 586 patients and found 12 with a falcine sinus, 1 of which was associated with a dysplastic tentorium. Three patients had an absent straight sinus, eight had a rudimentary straight sinus, and four had a normal straight sinus.

Another potential source of variation is the persistence and the functional role of embryological sinuses. The cerebellar tentorial sinuses were first identified by Gibbs and Gibbs during their study of the torcular Herophili and lateral sinuses [78,79,80,81,82]. Subsequent studies have detailed their anatomical and developmental variations. Developmentally, tentorial sinus positioning varies as cranial fossae and the temporal lobe expand. Duval et al. [83] examined 23 skulls and found the tentorial sinus in over half. Matsushima et al. [84] studied 26 tentorial sides in 13 specimens, classifying them into four groups:

- Group I (69.2%): drains cerebral hemisphere veins via bridging veins;
- Group II (88.5%): drains cerebellar hemispheric/vermian veins (subdivided further);
- Group III (42.3%): formed by small veins in the tentorium;
- Group IV (7.6%): formed by bridging veins from cerebral or brainstem structures.

Muthukumar and Palaniappan [85] found tentorial sinuses in 86% of 80 cadavers, categorizing them into the following types:

- Type I (25%): large, medial 1/3, draining into the straight sinus or torcular Herophili;
- Type II (25%): small, lateral 1/3, draining into the transverse–superior petrosal sinus junction;
- Type III (50%): medium sized, draining similarly to Type I.

Miabi et al. [86] classified lateral tentorial sinuses in 104 of 110 lobes into the following:

- Type I (28.8%): “candelabra” veins;
- Type II (21.1%): independent veins;
- Type III (35.5%): venous lakes.

Absent or accessory tributaries (e.g., occipital sinus or internal cerebral veins) are occasional anomalies [53].

The falcine sinus is typically an embryonic structure connecting the superior sagittal and straight sinuses. Tubbs et al. [87,88] found extensive networks within the falx cerebri in all 27 adult specimens, classifying communication with the superior sagittal sinus into the following:

- Type I (37%): no communication;
- Type II (26%): limited communication;
- Type III (37%): extensive communication.

Persistent falcine sinuses are linked to congenital anomalies like corpus callosum agenesis, Chiari malformation, or vein of Galen malformations.

The smallest venous sinus, the occipital sinus, originates at the foramen magnum and typically drains into the confluence of sinuses [89]. Variants include duplication, absence, or large sizes that replace other sinuses like the sigmoid sinus. Knott [52] found single and bilateral occipital sinuses in 42 of 44 specimens, with variable drainage patterns. Rollins et al. [90] reported on the age-related regression of occipital sinuses, which is found in 13% of children under 2 years but only 2% over 5 years.

Encircling the foramen magnum, the marginal sinus communicates with adjacent venous systems, such as the basilar and vertebral venous plexuses. Tubbs et al. [91,92] found consistent connections between the marginal sinus and hypoglossal canal structures in 93% of specimens.

#### 4.3.1. Superior Sagittal Sinus Variations

Several anatomic variations of the SSS (such as unilateral hypoplasia, aplasia of the rostral segment, duplication, or fenestration) are typical findings in cerebral angiograms [93].

Complete agenesis of the SSS is extremely rare and often associated with abnormalities of the falx cerebri due to their shared embryologic origin. Imaging modalities such as CT, MRI, and DSA reveal a lack of contrast filling in all segments of the SSS. Venous drainage in such cases is rerouted through enlarged compensatory venous structures, including the tentorial sinus, parasagittal large venous channels, and frontal hemispheric veins. These compensatory channels can sometimes mimic arteriovenous malformations or fistulas, leading to potential misdiagnosis [94,95].

Duplication or septation of the SSS typically occurs in its posterior portion and may drain into one or both TSs. This phenomenon is linked to incomplete fusion of the right and left primordia of the SSS during embryogenesis, persisting as a duplication. Duplication is sometimes associated with occipital or parietal meningoencephaloceles and anomalies in the course of the straight sinus [93,96].

Unilateral hypoplasia of the SSS, especially in its rostral third, is the most common anatomical variation after preferential TS drainage. In complete hypoplasia, the rostral portion is replaced by a pair of large parasagittal superior frontal cortical veins, which drain dorsally into the SSS at or near the coronal suture. Radiologic imaging, including DSA, CTV or MRV, may occasionally detect a rudimentary segment, although tributary cortical and meningeal veins are usually absent. Differentiating this condition from thrombosis of the anterior SSS requires identifying prominent compensatory frontal veins along the parasagittal course [95].

The prevalence of hypoplastic rostral SSS is 1.8–6% of cases, with compensatory venous drainage often occurring via intradural channels parallel to the midsagittal plane [97]. More pronounced hypoplasia, where the rostral SSS is entirely absent, is rarer. A study by Ruíz et al. [50] identified unilateral hypoplastic rostral SSS in 7% of cases, often accompanied by rerouted venous drainage through structures like the inferior sagittal sinus or interhemispheric frontal veins. In approximately 50.9% of the population, the SSS terminates midline at the internal occipital protuberance as a true sinus confluence. In the remaining cases, the SSS bifurcates above the internal occipital protuberance, with drainage occurring preferentially into the right TS twice as often as into the left [98].

Understanding these variations is critical for distinguishing normal anatomy from pathological conditions, such as thrombosis, and for accurately interpreting venographic imaging. Moreover, awareness of these anomalies aids in avoiding complications during surgical interventions involving the dural sinuses.

#### 4.3.2. Transverse Sinus Variations

The TSs exhibit notable anatomic asymmetry, a common and normal variation. Unilateral hypoplasia or aplasia has been observed in approximately 20–39% of individuals [99,100,101]. These variations underscore the diversity in venous drainage pathways within the cranium. AGs and fibrous trabeculae or septa are frequent findings within the TSs. A study by Strydom et al. [102] reported these structures in 53% of cases, with a predilection for the right TS. AGs are projections of the arachnoid membrane that extend into the sinus lumen, often creating filling defects visible on imaging. Similarly, fibrous trabeculae and septa appear as thin bands spanning the sinus lumen. The presence of trabeculae and septa within the TSs may provide structural support, preventing overdilation or collapse of the sinus walls. These features, while physiologically significant, can mimic pathological filling defects, such as those seen in venous thrombosis. Understanding their prevalence and characteristics is essential for the accurate interpretation of imaging studies.

Anatomic variations, including hypoplasia or aplasia, and intraluminal structures like AGs or trabeculae, can complicate the diagnosis of conditions like dural venous sinus thrombosis. Distinguishing between these normal variants and pathological findings is crucial. This asymmetry and structural diversity highlight the complex anatomy of the transverse sinuses, underscoring the need for detailed imaging and careful analysis in clinical practice [102]. The main variations of the TS are summarized in Table 5.

Asymmetry and variations can mimic venous thrombosis on imaging, necessitating careful evaluation. Flow gaps or filling defects caused by hypoplasia, aplasia, or septa are commonly misinterpreted. Variations can affect results of the Tobey–Ayer–Queckenstedt test used in diagnosing sinus thrombophlebitis.

## 5. Intracranial Sinuses Stenosis

Stenosis of the dural venous sinuses is frequently observed in patients with idiopathic intracranial hypertension (IIH). Elder et al. [103] reported that the incidence of transverse sinus stenosis in IIH patients ranges from 10% to 90%, compared to 6.8% in the general population. Similarly, Farb et al. [104] found that 93% of IIH patients exhibited bilateral venous sinus stenosis on MRV, compared to only 7% of controls. While the precise etiology of venous sinus stenosis in IIH—whether intrinsic or extrinsic—remains debated, endovascular stenting has emerged as a promising treatment option for patients with medically refractory IIH. This approach has shown greater efficacy in managing symptoms and providing longer-lasting relief compared to earlier interventions. The transverse venous sinuses are susceptible to collapse in the context of elevated intracranial pressure (ICP), making them prone to extrinsic stenosis, which is characterized by smooth, long tapering. This type of stenosis often resolves with a reduction in IIH. In contrast, intrinsic stenosis is caused by fixed intraluminal structures that disrupt venous flow dynamics. These include fenestrations, arachnoid granulations, organized chronic thrombus, and embryological remnants such as septations. Unlike extrinsic stenosis, intrinsic stenosis does not improve with a reduction in CSF pressure. Arachnoid villi are semipermeable protrusions of the subarachnoid space into the cerebral venous sinuses, facilitating passive CSF drainage based on a pressure gradient. When enlarged, they are referred to as arachnoid granulations (AGs), mushroom-shaped structures that can potentially obstruct the sinus lumen. Strydom et al. [102] reported AGs in 30% of cases (based on cadaver studies and post-contrast CT imaging), with a higher prevalence in the right transverse sinuses, often coexisting with septations. AGs are believed to increase in both size and number with age and may eventually play a role in the pathophysiology of IIH [105,106].

The venous sinus is a unique anatomical structure formed by dural folds. Unlike typical veins, it lacks venous valves and the usual vascular wall layers (intima, media, and adventitia) [107]. One notable variation associated with this structure is the dural sinus septum, believed to result from the merging and formation of the embryonic venous plexus during development [108]. First identified in a 1975 autopsy study, the incidence of straight sinus septa was reported at approximately 20% [109]. Subsequent autopsy research has indicated an even higher incidence, with rates reaching up to 44% [102]. The dural sinus septum can present specific imaging characteristics, such as a filling defect or a double-lumen sign on postmortem radiography, which are important to distinguish from venous sinus thrombosis. Modern imaging modalities like high-resolution MRI, three-dimensional CT venography, and intravascular ultrasound have also confirmed its presence [20,60,110]. In a study aiming to assess the presence of dural sinus septum as cause for TS stenosis in patients with intracranial hypertension, DSA was employed, defining the presence of a sinus septum as double-lumen appearance on angiography [111]. The authors found an occurrence rate of 17.3%, which is lower than anatomical studies, likely due to the limitations of DSA in detecting small septa. While the existence of dural sinus septa is well established, their pathophysiological effects remain a subject of debate. Some studies consider the septa to be physiological structures that stabilize the venous sinus, preventing dilation or collapse. For instance, Strydom et al. suggested that torn septa might form valvular structures, potentially obstructing normal cerebral venous drainage and increasing sinus venous pressure [102]. Conversely, other research views dural sinus septa as pathological. Subramaniam et al. [112] found that septa could alter venous hemodynamics, leading to stenosis or thrombosis. Additionally, in a study of patients with idiopathic intracranial hypertension, 23.1% had septa in the transverse sinus, implicating these structures in the disease’s pathology [113]. Transverse sinus septa have been divided in three types based on their angiographic appearance:

- Type I: this type has the highest incidence and often exhibit a strong double-lumen sign on DSA.
- Type II: the second most common type: it is smaller in size and presents with a weaker imaging signal.
- Type III: the rarest type: it has less well-defined anatomical characteristics.

Beyond the etiopathogenetic role of TS stenosis and dural sinus stenosis in general in idiopathic intracranial hypertension, whether they are the first moments or amplifiers of a damage mechanism, their anatomical characteristics make them superimposable as potential mimics of CVT to what is described in the previous sections. In addition, for some stenoses, the nature of partial recanalization of previous thrombosis has also been hypothesized.

**Figure 10:**
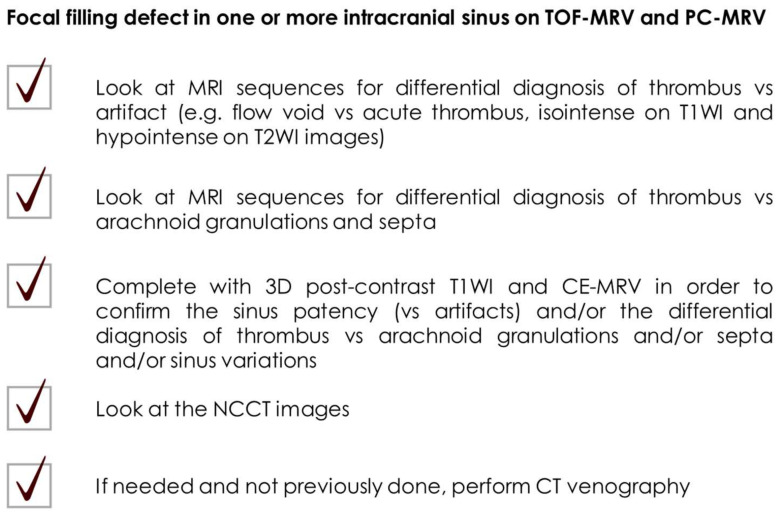
Pragmatical checklist of caveats in interpreting the focal filling defects of intracranial venous sinuses on TOF-MRV and PC-MRV.

A checklist of the main caveats in interpreting thrombotic vs. non-thrombotic filling defects in intracranial sinuses is illustrated in Figure 10.

## 6. Conclusions

Non-thrombotic filling defects of the dural venous sinuses can have multiple origins, from artefactual genesis (in relation to the characteristics of the focal flow and the MRI technique used) to anatomical anomalies up to the presence of, within the natural sinus, normal anatomical structures, such as arachnoid granulations and septa or chordae Willisii. The differential diagnosis of these structures, with respect to a thrombotic and sometimes articulated occlusion, but with knowledge of the anatomical characteristics of these structures and that of the available investigation techniques, allows, in most cases, for the resolution of the diagnostic question, at which point it is often necessary to use different neuroradiological techniques to be conclusive in the differential diagnosis between CVT and non-thrombotic formations that act as flow defects in the images. This distinction is fundamental because the diagnosis of CVT implies a medical treatment not free from risks.

## Figures and Tables

**Table 1:** Main features of MR venography techniques.

Issue	TOF	PC	CE-MRV
Physical principle	Flow enhancement	Phase shift	T1 shortening
Sequence	Gradient echo-based	Gradient echo-based	Gradient echo-based
GBCA	No	No	Yes
Background	Partly suppressed	Suppressed	Enhancing lesions seen
Pitfalls	Saturation of exogenous signal (arteries)	Operator for selection of appropriate VENC	Scan timing for maximum venous enhancement
Flow velocity	Can detect slow flow	Depends on the VENC	Independent
Flow direction	Not detected	Detected	Not detected
Flow quantification	No	Yes	No

**Table 2:** Evolution of thrombus signal on MRI [18].

Time	Hemoglobin Degradation Product	T1WI	T2WI and FLAIR
<5 days	Deoxyhemoglobin	Iso/hypointense to brain tissue	Iso/hypointense to brain tissue
6–14 days	Methemoglobin	Hyperintense	Hyperintense
>14 days	(Fibrosis/recanalization)	Variable	Variable

**Table 3:** Classification of chordae Willisii [21,22].

Type	Features
Valvelike chordae	These are the most common type, constituting approximately 45–49% of all recorded chordae. Typically found near the lateral sinus wall, they cover the openings of the superior cerebral veins and prevent blood backflow. This design ensures unidirectional drainage from the veins into the sinus. Valvelike chordae are particularly concentrated in the middle 12–14 cm of the SSS, where venous orifices often enter at physiologically unfavorable angles. They are less frequent at the anterior 5–7 cm of the SSS.
Trabecular chordae	These comprise 30–35% of the chordae and cross the lumen at varying angles, either centrally or peripherally. Trabecular chordae often appear as isolated strands, small clusters, or networks. They contribute structural support and regulate blood flow dynamics. Most trabecular chordae are unrelated to afferent veins, with asymmetry favoring more intense blood flow on the right side of the SSS.
Longitudinal lamellae	These chordae account for 20–23% of cases and typically divide the SSS lumen into two or three distinct channels. Their triangular or square morphology with concave frontal and occipital borders helps support the sinus lumen, preventing compression and maintaining pressure equilibrium. Their length ranges from 2 to 35 mm, although most are within 2–8 mm.
Accessory cords [23]	They constitute 1.6% of structures within the SSS. These cordlike formations often form networks at venous lacunae entrances, further aiding in the regulation of venous drainage.

**Table 4:** Classifications of torcular Herophili variations.

Proponent	Descriptions	
Woodhall [58]	Plexiform type (56%)	Multiple channels communicate between the sides.
	Ipsilateral type (31%)	The SSS drains toward one side, and the straight sinus drains toward the opposite side.
	Common pool type (9%)	The SSS and straight sinuses merge, draining into both TSs.
	Unilateral type (4%)	Both the SSS and straight sinuses drain into the same TS, with the contralateral TS absent.
	Occipital type	A large single sinus or paired occipital sinuses persist with a large marginal sinus.
Browning [59]	Type I (36%)	The SSS and straight sinuses merge into a confluence, from which both TSs arise.
	Type II (24%)	Both the SSS and straight sinuses bifurcate and their right and left branches unite to form the corresponding TSs.
	Type III (24%)	The SSS deviates to the right of the left TS, while the straight sinus bifurcates into two branches: one joining the SSS and the other becoming the opposite TS.
	Type IV (16%)	This pattern is similar to Type III, but the drainage of the straight and sagittal sinuses is reversed.
Bisaria [48,53]	Type 1 (24.5%)	The SSS drains into one TS, and the straight sinus drains into the other.
	Type 2 (9.1%)	Both the SSS and straight sinuses bifurcate, draining into the respective TSs.
	Type 3 (57.3%)	The torcular Herophili is present, with varying degrees of partitioning.
Fukusumi [60]	Type I (20%)	The SSS drains into the torcular Herophili, which bifurcates into two TSs.
	Type II (26.7%)	The SSS splits into right and left limbs, draining into the same side of the TS.
	Type III (44.2%)	The SSS drains completely into the right TS.
	Type IV (9.2%)	The SSS drains completely into the left TS.
Singh [61]	Confluence type (35%)	The SSS drains into a common venous pool.
	Bifurcation type (14%)	The SSS divides into bilateral TSs.
	Right-dominant type (41%)	The SSS drains only into the right TS.
	Left-dominant type (10%)	The SSS drains only into the left TS.
Kobayashi [62]	Type I (32.7%)	The superior sagittal, straight, and TSs merge.
	Type II	The SSS bifurcates into: a. Right TS (31.5%) b. Left TS (6.2%)
	Type III	The SSS and straight sinus branch into bilateral TSs (18.9%).
	Type IV	The straight sinus branches into the left and right TSs: a. Right TS (3.1%) b. Left TS (4.4%)
	Type V	a. The straight sinus drains into the occipital sinus (0.5%). b. The straight sinus drains into other veins (0.4%).
	Type VI	Aplasty of the TSs: a. Left TS (0.4%) b. Right TS (0.9%)
	Type VII	The straight sinus branches into bilateral TSs, and the SSS drains into the straight and left TSs (0.2%).
	Type VIII	The SSS branches into bilateral TSs, while the straight sinus branches into the SSS and left TS (0.4%).
	Type IX	The SSS branches into the right TS and occipital sinus. The straight sinus drains into the left TS and occipital sinus, with an anastomosis between the straight and SSS (0.4%).

**Table 5:** Transverse sinus variations [44].

Issue	Features
Asymmetry and dominance	Asymmetry is a common feature, with the right TS typically larger and more dominant than the left. Right dominance: 59–62% of cases Left dominance: 25–30% of cases Codominance: 10–16% Hypoplasia or aplasia: Left hypoplasia: 39% of cases Left aplasia: 20% of individuals Right hypoplasia or aplasia: much rarer, affecting only 4–6% of cases.
Fibrous septa, bands, and doubling	Septa or fibrous bands can be present within the sinus, occasionally narrowing the lumen. Doubling of the TS has been observed, more commonly on the left. These structures may create flow gaps, often being mistaken for pathologies like venous thrombosis.
Connections and communications	The TS may have multiple openings connecting to the torcular Herophili. Rarely, an oblique sinus can connect the left and right TSs. Hypoplasia or aplasia of one TS often leads to compensatory enlargement of the contralateral sinus. In cases where the TS is absent, drainage is rerouted via the occipital sinus, marginal sinus, or superior petrosal sinus.
Developmental variations	Age-related changes: hypoplasia or aplasia may appear more frequently in older individuals due to changes in cranial venous flow. Overlapping lambdoidal sutures: in infants, these sutures may cause septation of the transverse sinus. Enlargement of structures: some individuals exhibit dilation of the distal TS, sigmoid sinus, or superior jugular bulb.
Rare and aberrant drainage patterns	Mastoid foramen drainage: Some cases show blood exiting the skull via the mastoid foramen rather than the jugular foramen. These cases may have an absent or hypoplastic sigmoid sinus. Alternative venous routes: The TS may connect with the cavernous sinus or ophthalmic veins via aberrant veins.

## Data Availability

Not applicable.
